# IL6 genotype, tumour ER-status, and treatment predicted disease-free survival in a prospective breast cancer cohort

**DOI:** 10.1186/1471-2407-14-759

**Published:** 2014-10-11

**Authors:** Andrea Markkula, Maria Simonsson, Christian Ingvar, Carsten Rose, Helena Jernström

**Affiliations:** Division of Oncology and Pathology, Department of Clinical Sciences, Lund, Lund University, Barngatan 2B, Lund, SE 22185 Sweden; Division of Surgery, Department of Clinical Sciences, SE-22185 Lund, Lund University and Skåne University Hospital, Lund, Sweden; CREATE Health and Department of Immunotechnology, Lund University, Medicon Village, Building 406, Lund, S-22381 Sweden

**Keywords:** Breast cancer, *IL6*, Oestrogen receptor, Chemotherapy, Radiotherapy, Treatment resistance

## Abstract

**Background:**

In breast cancer, high levels of the inflammatory cytokine interleukin-6 (IL-6) have been associated with disease-free survival and treatment resistance. Increased serum levels of IL-6 have been correlated with increased levels of NF-κβ and aromatase expression in adipose tissue. Several *IL6* single nucleotide polymorphisms have been associated with breast cancer prognosis, but the impact may differ depending on tumour oestrogen receptor (ER) status. This translational study investigated the association between *IL6* genotypes, ER-status, and treatment on the risk of early events among breast cancer patients.

**Methods:**

The study included 634 25- to 99-year-old primary breast cancer patients in Sweden from 2002–2008. Genotyped *IL6* single nucleotide polymorphisms rs1800797, rs1800796, rs1800795, and rs2069849 were analysed separately and as diplotypes. Disease-free survival was assessed for 567 patients. Clinical data, patient-, and tumour-characteristics were obtained from questionnaires, patient charts, population registries, and pathology reports.

**Results:**

The median follow-up time was 5.1 years. *IL6* diplotype was not associated with early events for all 567 patients, but AGCC/AGCC diplotype-carriers with ER-negative tumours had an increased risk, (adjusted Hazard Ratio (HR) = 5.91, 95% CI: 1.28–27.42). Any C-carriers (rs1800795) with ER-negative tumours had a higher risk of early events than GG-carriers with ER-negative tumours, (adjusted HR = 3.76, 95% CI: 1.05–13.43), particularly after radiotherapy (adjusted HR = 7.17, 95% CI: 1.16–32.28). Irrespective of ER-status, chemotherapy-treated Any C-carriers had a higher risk of early events than GG-carriers (adjusted HR = 3.42, 95% CI: 1.01–11.54).

**Conclusions:**

The main finding of the present study was that *IL6* genotype was strongly associated with early events among patients with ER-negative tumours, particularly among radiotherapy-treated patients, and among chemotherapy-treated patients irrespective of ER-status. The high risk for early events observed in these subgroups of patients suggests that combined information on *IL6* genotype, tumour ER-status, and breast cancer treatment may represent a tool for identifying patients who require more personalised treatment.

**Electronic supplementary material:**

The online version of this article (doi:10.1186/1471-2407-14-759) contains supplementary material, which is available to authorized users.

## Background

Breast cancer is the most prevalent type of cancer among women and the primary cause of cancer death among women worldwide [1]. Breast cancer treatment resistance is common and increases mortality [2]. Novel prognostic and treatment-predictive markers may lead to more personalised breast cancer treatment and improved prognosis.

In breast cancer patients, a high level of the inflammatory cytokine interleukin-6 (IL-6) has been associated with increased tumour stage, lymph node infiltration, recurrence, and treatment resistance [3–5]. Increased serum levels of IL-6 has been correlated with increased levels of NF-κβ, which may represent a mechanism for tumour resistance to chemotherapy and radiotherapy [6]. Further, IL-6 has been shown to stimulate aromatase expression in adipose tissue; aromatase expression subsequently stimulates oestrogen synthesis, potentially contributing to breast cancer progression [7].

Several *IL6* single nucleotide polymorphisms (SNPs) have been associated with breast cancer risk and prognosis. An *IL6* haplotype that consists of the SNPs rs1800797 (-596A > G), rs1800796 (-572G > C), -373 [10A/11 T], and rs1800795 (-174G > C) was associated with reduced disease-free survival among breast cancer patients [8]. In a previous study, postmenopausal women with the SNP rs1800797 AA genotype had an increased risk of breast cancer, even if they had not recently been exposed to hormones [9]. Further, patients with oestrogen receptor (ER)-positive tumours and the SNP rs1800797 GG genotype had reduced disease-free survival compared to patients with the CC or GC genotypes [8].

The well-studied SNP rs1800795/-174 G > C that is located in the promoter region of *IL6* has been associated with fatigue and survival among breast cancer patients [8, 10–12], and the effects of this SNP appear to vary according to tumour ER-status [8, 10]. The relationship between genotype and plasma levels of IL-6 appears to be complex. The -174 C-allele has been associated with increased IL-6 and C-reactive protein (CRP) levels, particularly in inflammatory conditions [13–15]; however, conflicting results have been reported [16, 17].

High levels of circulating IL-6 are associated with fatigue and depression among breast cancer patients [6], and depression is associated with reduced breast cancer survival [18]. Antidepressant treatment reduced IL-6 levels in depressed patients [19] and increased adherence to adjuvant breast cancer treatment [20]. In addition, antidepressant treatment has been hypothesised to increase survival among depressed cancer patients [21].

To identify novel breast cancer markers of possible prognostic or treatment-predictive importance among breast cancer patients, investigations of the combined effects of antidepressant use, breast cancer treatment, ER-status, and *IL6* genotype on breast cancer prognosis are necessary. We hypothesised that *IL6* genotype can affect the risk of early events and treatment response and that the impact of this genotype is further modified by ER-status. Hence, the aim of this study was to investigate the impact of the *IL6* SNPs rs1800797, rs1800796, rs1800795, rs2069849 and *IL6* diplotypes based on these SNPs in relation to tumour ER-status on early events and treatment response.

## Methods

### Study population

Beginning in October 2002, women who were diagnosed with a first breast cancer at the Skåne University Hospital in Lund, Sweden were invited preoperatively to participate in the ongoing prospective BC-blood study. The Skåne University Hospital in Lund serves nearly 300,000 inhabitants. Since patients are not referred to other hospitals for surgery, the cohort is considered population-based. During the time the cohort was compiled, 1,090 patients received breast cancer surgery at the hospital. Approximately 58% of these patients were included in the study. Patients were missed primarily due to a lack of available research nurses. The included patients were similar to the non-included patients with respect to age and hormone receptor status [22]. Patients with a prior history of breast cancer or another cancer diagnosis within the previous ten years were excluded. The majority of the patients who were diagnosed in Lund were ethnic Swedes; however, ethnicity information was not obtained during this study.

This paper presents data collected from 634 patients who initiated treatment between October 2002 and October 2008. Treatment was administered according to the standard of care at Skåne University Hospital. The patients were asked to complete questionnaires prior to surgery, three to six months after surgery and one, two, three, five, seven, and nine years after surgery. The follow-up rates in the present cohort were high [23]. Written informed consent was obtained from all patients, and the study was approved by the Lund University ethics committee (Dnr 75–02, 37–08, and 658–09).

During the preoperative visit, blood samples were collected for genotyping. The research nurses also measured body weight, height, and waist and hip circumferences during the preoperative visit. The volume of each breast was measured using plastic cups, as previously described [24]. The waist circumference was measured at the umbilicus; the hip circumference was measured at the widest part between the hip and trochanter major. The questionnaire included questions regarding the date of surgery, reproductive history, exogenous hormone use, smoking history (i.e., yes/no/occasional smoker) and alcohol consumption. Patients who identified themselves as regular smokers and occasional smokers during the preoperative visit or at any subsequent visit were classified as smokers. A body mass index (BMI) cut-off value of 25 kg/m^2^ was used, according to the WHO’s classification of overweight [25]. Central obesity was considered to be present if the waist-to-hip ratio (WHR) was above 0.85 [25]. Questions regarding alcohol consumption frequency were based on the alcohol use disorders identification test (AUDIT) [26]. A breast volume cut-off of 850 ml was chosen based on a previous publication [27]. Mammography-detected tumours in patients aged 45–74 years at the time of diagnosis were considered to be screening-detected. Patients within this age category were invited to mammography screening in Sweden during the study inclusion period. Antidepressant use was coded as a dummy variable based on the information obtained from the preoperative questionnaire [28].

Information regarding the type of adjuvant treatment, sentinel node biopsy results, axillary lymph node dissection and type of surgery was collected from patient charts. Treatment information was also collected from questionnaires and was recorded up to the time of the last follow-up appointment or death, prior to any event. Data on invasive tumour size, histological type and grade, and number of involved axillary lymph nodes were obtained from each patient’s pathology report. ER and progesterone receptor (PgR) status were determined as previously described [29, 30].

The tumours were analysed in the Department of Pathology at Skåne University Hospital in Lund. Information concerning breast cancer events, including local or regional recurrence, new breast cancer, or distant metastases, was obtained from patient charts, pathology reports and the Regional Tumour Registry. The date of death was obtained from the Swedish Population Registry.

### Genotyping

Genomic deoxyribonucleic acid (DNA) was extracted from the leukocyte portion of whole blood using a Wizard Genomic DNA Purification Kit (Promega, Madison, WI, USA). Genotyping was performed at the Region Skåne Competence Centre (RSKC Malmö) of Malmö University Hospital in Malmö, Sweden. The SNPs rs1800797, rs1800796, rs1800795, and rs2069849 were analysed via matrix-assisted laser desorption/ionisation time-of-flight mass spectrometry using a Sequenom MassARRAY® platform (Sequenom, San Diego, CA, USA) and iPLEX reagents, according to the manufacturers’ protocol. Sequenom MassARRAY® software (Sequenom) was used for multiplex SNP analysis design. Over 10% of the samples were run in duplicate, with a concordance of 100%.

### IL6 diplotype construction

Each SNP was cross-tabulated against the other three SNPs. This procedure demonstrated that certain combinations did not exist or were very rare. Therefore, we constructed the haplotypes and diplotypes based on the most likely combinations. The *IL6* diplotype consisted of four *IL6* SNPs: rs1800797 (-596A > G), rs1800796 (-572G > C/-634C > G), rs1800795 (-174 G/C), and rs2069849 (C/T coding exon 5). Diplotype variants that were present in less than 5% of the patients were classified as rare variants and combined into a single group termed ‘rare diplotypes.’ *IL6* diplotype information was missing for nine patients. Rs1800797 analysis failed for five patients; of these cases, three SNPs could be imputed based on rs1800795, as the R^2^ between these two SNPs was 0.966. Consequently, while rs1800795 analysis failed for four patients, two SNPs could be imputed based on rs1800797. The two remaining SNPs lacked information related to both Rs1800797 and Rs1800795 and could therefore not be imputed. Rs1800796 analysis failed for seven patients.

### Data analyses

Statistical analyses were performed using IBM SPSS Statistics 19.0 (Chicago, IL, USA). Each patient’s BMI was calculated by dividing weight in kilograms by the square of patient height in meters (kg/m2). The WHR was calculated as waist circumference divided by hip circumference.

*IL6* genotype was analysed in relation to tumour and patient characteristics. *IL6* gentype was analysed in relation to patient characteristics (i.e., age at diagnosis, weight, height, BMI, WHR, age at menarche, and total breast volume) using the non-parametric Kruskal-Wallis test because these variables were continuous and not normally distributed. Chi-square analyses were used to investigate the relationship between *IL6* genotype and the categorical variables breast volume ≥850 ml (yes/no), parous (yes/no), preoperative use of antidepressants (yes/no), any hormone replacement therapy use (HRT) (yes/no), preoperative smoking (yes/no), smoking at any visit (yes/no), alcohol consumption frequency, screening-detected tumour (yes/no), invasive tumour size (in situ, ≤20 mm, 21–50 mm, ≥51 mm, skin or muscle involvement, or 21 mm or larger), histological grade (I-III or III), axillary lymph node involvement (0, 1–3, 4+, or any axillary lymph node involvement (yes/no)), ER-status (positive/negative), PgR status (positive/negative), and the combination variables ER- and PgR-positive (yes/no), ER- and PgR-negative (yes/no), ER-positive and PgR-negative (yes/no), and ER-negative and PgR-positive (yes/no).

To analyse breast cancer-free survival, patients were followed from inclusion to the first breast cancer event. Patients without events were followed until the last follow-up or death prior to January 1, 2013. Of 634 patients, 567 were included in the survival analyses of *IL6* diplotype and 574 patients were included in the survival analyses of rs1800795.

For the univariable survival analysis, the Log-Rank test was used to analyse the risk of early cancer events in relation to *IL6* genotype, ER-status, and breast cancer treatment. Due to the small number of homozygous rs1800795 minor allele carriers, the G/C and C/C genotypes were combined into a single “Any C” genotype for the survival analyses. For the multivariable analysis, Cox regression was used to calculate Hazard Ratios (HRs) in relation to rs1800795, adjusting for age (linear), invasive tumour size (≥21 mm or muscular or skin involvement), axillary lymph node involvement (yes/no), and histological grade III (yes/no). Since few patients exhibited an invasive tumour size ≥51 mm or muscular or skin involvement, these patients were combined with the patients with invasive tumour sizes between 21 and 50 mm in the multivariable analyses. In the multivariable analyses of rs1800795, a categorical variable that combined rs1800795 and ER-status was used. Adjustments were made using breast volume, BMI, and WHR as either dichotomous or continuous variables. Breast volume was not normally distributed and was therefore transformed using the natural logarithm (ln). Prior power calculations that assumed 600 patients with an accrual interval of 6 years, an additional follow-up time of 2 years and 30% of patients with a variant allele demonstrated that the study was able to detect true HRs between 0.722 and 1.440 with 80% power and an α of 5% [31]. Further, simulations with 80% failure rates were also performed, demonstrating that the study had sufficient power to detect an increased HR of 1.9 with a genotype frequency of 30%.

A *P*-value <0.05 was considered significant. All *P*-values were two-tailed. Since this was an exploratory study, nominal *P*-values are presented without adjustments for multiple testing. The report is based on the REMARK criteria [32].

## Results

*IL6* htSNP analyses were performed for 634 patients. Genotype information was missing for nine women. Five different haplotypes and 11 different diplotypes were identified in the cohort. The two most common haplotypes were AGCC (45.3% of patients) and GGGC (46.6% of patients). The three most common diplotypes were AGCC/GGGC, AGCC/AGCC, and GGGC/GGGC (38.2, 23.2, and 23.5% of patients, respectively). The eight diplotype variants that were present in less than 5% of the patients were classified as rare variants and combined into a single group termed ‘rare diplotypes’.

### Patient and tumour characteristics

Patient characteristics for the 567 patients with diplotype information and the 574 patients with information on rs1800795 genotype who were included in the survival analyses are presented in Table 1. Tumour characteristics for the 567 patients with diplotype information and the 574 patients with information on rs1800795 genotype who were included in the survival analyses are presented in Table 2. Patient characteristics for all 634 patients, for the 567 patients with diplotype information, and the 574 patients with information on rs1800795 genotype who were included in the survival analyses are presented in Additional file 1. Tumour characteristics for all patients included the 592 patients who had not received preoperative treatment, for the 567 patients included in the diplotype survival analyses, and for the 574 patients included in the rs1800795 survival analyses are presented in Additional file 2. A borderline significant association was observed between carrying the AGCC/GGGC diplotype and having a non-screening-detected tumour (*P* = 0.063). The GGGC/GGGC diplotype was borderline significantly associated with ER and PgR negativity (*P* = 0.055). For rs1800795, GC-carriers had a borderline significant increased risk of an invasive tumour size ≥21 mm (*P* = 0.061).

**Table 1:** **Patients included in the survival analyses stratified according to IL6 diplotype (A) and rs1800795 (B)**

	(A)						(B)				
	Patients included in the diplotype survival analyses		Stratified according to IL6 diplotype*				Patients included in the rs1800795 survival analyses		Stratified accoding to rs1800795**		
			GGGC/GGGC	AGCC/GGGC	AGCC/AGCC	Rare diplotypes			GG	GC	CC
	Median (IQR***) or n (%)	Missing	Median (IQR) or n (%)	Median (IQR) or n (%)	Median (IQR) or n (%)	Median (IQR) or n (%)	Median (IQR***) or n (%)	Missing	Median (IQR) or n (%)	Median (IQR) or n (%)	Median (IQR) or n (%)
**n=**	567		130 (22.9)	221 (39.0)	131 (23.1)	85 (15.0)	574		181 (31.5)	257 (44.8)	136 (23.7)
**Age at diagnosis, yrs**	59.7 (51.8-66.3)	-	60.5 (49.2-66.7)	59.5 (51.4-66.7)	59.8 (53.8-66.4)	58.4 (51.1-65.2)	59.8 (52.0-66.4)	-	60.5 (49.9-67.1)	59.5 (52.2-66.4)	59.8 (53.8-66.3)
**Weight, kgs**	68.0 (61.0-76.7)	2	69.0 (62.0-81.3)	67.0 (60.0-75.0)	68.0 (61.0-78.5)	68.3 (62.0-77.6)	68.0 (61.0-76.9)	2	69.0 (61.9-80.0)	67.1 (60.4-75.0)	69.0 (61.0-78.9)
**Height, m**	1.66 (1.62-1.70)	1	1.66 (1.62-1.71)	1.65 (1.62-1.69)	1.65 (1.62-1.70)	1.67 (1.60-1.70)	1.66 (1.62-1.70)	1	1.66 (1.60-1.71)	1.66 (1.62-1.69)	1.66 (1.62-1.70)
**BMI, kgs/m** ^**2**^	24.6 (22.3-27.7)	3	25.2 (22.9-29.1)	24.3 (21.9-26.9)	24.6 (22.4-28.6)	24.4 (22.2-28.2)	24.6 (22.3-27.9)	3	25.2 (22.6-28.7)	24.4 (22.1-27.4)	24.6 (22.4-28.8)
**Breast volume**	1000 (600–1450)	75^a^	1000 (700–1538)	950 (600–1450)	988 (600–1450)	900 (585–1563)	1000 (600–1450)	75^a^	950 (650–1400)	1000 (625–1563)	1000 (600–1450)
**Breast volume ≥850 ml**	281 (57.1)	75^a^	68 (60.7)	110 (55.6)	65 (59.1)	38 (52.8)	286 (57.3)	75^a^	86 (55.8)	132 (57.4)	68 (59.1)
**Waist-Hip Ratio**	0.84 (0.78-0.89)	2	0.84 (0.78-0.90)	0.83 (0.78-0.88)	0.84 (0.79-0.89)	0.83 (0.78-0.89)	0.84 (0.78-0.89)	2	0.83 (0.78-0.90)	0.83 (0.78-0.88)	0.84 (0.79-0.89)
**Age at menarche, yrs**	13 (12–14)	3	13 (12–14)	13 (12–14)	14 (13–14)	13 (12–14)	13 (12–14)	3	13 (12–14)	13 (12–14)	14 (13–14)
**Parous**	485 (85.5)	-	111 (85.4)	192 (86.9)	108 (82.4)	74 (87.1)	490 (85.4)	-	156 (86.2)	222 (86.4)	112 (82.4)
**Alcohol use**		1						1			
Never	62 (11.0)		19 (14.6)	23 (10.4)	15 (11.5)	5 (6.0)	62 (10.8)		23 (12.8)	24 (9.3)	15 (11.0)
Not more than once a month	149 (26.3)		37 (28.5)	57 (25.8)	34 (26.0)	21 (25.0)	154 (26.9)		48 (26.7)	69 (26.8)	37 (27.2)
2-4 times per month	216 (38.2)		41 (31.5)	84 (38.0)	54 (41.2)	37 (44.0)	217 (37.9)		64 (35.6)	97 (37.7)	56 (41.2)
2-3 times per week	110 (19.4)		27 (20.8)	51 (23.1)	19 (14.5)	13 (15.5)	111 (19.4)		34 (18.9)	58 (22.6)	19 (14.0)
4 or more times per week	29 (5.1)		6 (4.6)	6 (2.7)	9 (6.9)	8 (9.5)	29 (5.1)		11 (6.1)	9 (3.5)	9 (6.6)
**Pre-operative smoker**	121 (21.3)	-	22 (16.9)	48 (21.7)	30 (22.9)	21 (24.7)	121 (21.1)	-	33 (18.2)	57 (22.2)	31 (22.8)
**Smoker at any visit**	129 (22.8)	-	23 (17.7)	51 (23.1)	32 (24.4)	23 (27.1)	130 (22.6)	-	36 (19.9)	60 (23.3)	34 (25.0)
**Pre-operative use of antidepressants**	56 (9.9)	-	14 (10.8)	21 (9.5)	16 (12.2)	5 (5.9)	57 (9.9)	-	18 (9.9)	23 (8.9)	16 (11.8)
**Ever use of HRT, %**	263 (46.5)	1	54 (41.9)	102 (46.2)	70 (53.4)	37 (43.5)	267 (46.6)	1	77 (42.8)	118 (45.9)	72 (52.9)
**Screening detected tumor**	289 (60.3)	88^b^	59 (56.7)	102 (54.8)	78 (68.4)	50 (66.7)	293 (60.5)	90^b^	84 (56.8)	129 (59.2)	80 (67.8)

*9 patients lacked genotype information.

**2 patients lacked genotype information.

***IQR Interquartile range.

^a^Analysis included women who had not gone through breast surgery before diagnosis.

^b^Analysis included women 45–74 years at diagnosis due to previous Swedish screening protocols.

**Table 2:** **Tumour characteristics for all patients included in the survival analyses, and stratified according to IL6 diplotype (A) and rs1800795 (B) (number of patients who had not received preoperative treatment indicated in boldface)**

	(A)						(B)				
	Patients included in the diplotype survival analyses		Stratified according to IL6 diplotype*				Patients included in the rs1800795 survival analyses		Stratified accoding to rs1800795**		
			GGGC/GGGC	AGCC/GGGC	AGCC/AGCC	Rare diplotypes			GG	GC	CC
	Median (IQR***) or n (%)	Missing	Median (IQR) or n (%)	Median (IQR) or n (%)	Median (IQR) or n (%)	Median (IQR) or n (%)	Median (IQR***) or n (%)	Missing	Median (IQR) or n (%)	Median (IQR) or n (%)	Median (IQR) or n (%)
	567		130 (22.9)	221 (39.0)	131 (23.1)	85 (15.0)	574		181 (31.5)	257 (44.8)	136 (23.7)
**Preoperative interstitial laser thermotherapy**	10 (1.7)		3 (2.1)	4 (1.7)	2 (1.5)	1 (1.1)	10 (1.6)		4 (2.1)	4 (1.5)	2 (1.4)
**Information on preoperative treatment missing**	1		0	0	0	1	1		0	1	0
**Neoadjuvant therapy**	26 (4.3)		8 (5.7)	9 (3.9)	5 (3.6)	4 (4.4)	26 (4.3)		9 (4.6)	12 (4.4)	5 (3.5)
**No preoperative treatment**	**567**		**130 (22.9)**	**221 (39.0)**	**131 (23.1)**	**85 (15.0)**	**574**		**181 (31.5)**	**257 (44.8)**	**136 (23.7)**
**Invasive tumour size (pT)**		-						-			
*≥21 mm (≥2)*	150 (26.5)		28 (21.5)	69 (31.2)	30 (22.9)	23 (27.1)	151 (26.3)		40 (22.1)	80 (31.1)	31 (22.8)
In Situ	-		-	-	-	-	-		-	-	-
≤20 mm (1)	417 (73.5)		102 (78.5)	152 (68.8)	101 (77.1)	62 (72.9)	423 (73.7)		141 (77.9)	177 (68.9)	105 (77.2)
21-50 mm (2)	141 (24.9)		27 (20.8)	64 (29.0)	27 (20.6)	23 (27.1)	142 (24.7)		39 (21.5)	75 (29.2)	28 (20.6)
51- mm (3)	8 (1.4)		1 (0.8)	4 (1.8)	3 (2.3)	0	8 (1.4)		1 (0.6)	4 (1.6)	3 (2.2)
skin or muscle involvement (4)	1 (0.2)		0	1 (0.5)	0	0	1 (0.2)		0	1 (0.4)	0
**Axillary node involvement**		2						2			
*Any lymph node involvement*	217 (38.4)		41 (31.8)	89 (40.3)	50 (38.5)	37 (43.5)	221 (38.6)		62 (34.4)	108 (42.0)	51 (37.8)
0	348 (61.6)		88 (68.2)	132 (59.7)	80 (61.5)	48 (56.5)	351 (61.4)		118 (65.6)	149 (58.0)	84 (62.2)
1-3	163 (28.8)		29 (22.5)	70 (31.7)	36 (27.7)	28 (32.9)	167 (29.2)		46 (25.6)	84 (32.7)	37 (27.4)
4+	54 (9.6)		12 (9.3)	19 (8.6)	14 (10.8)	9 (10.6)	54 (9.4)		16 (8.9)	24 (9.3)	14 (10.4)
**Histological grade**		1						1			
*Grade III*	111 (19.6)		30 (23.1)	49 (22.3)	18 (13.7)	14 (16.5)	113 (19.7)		37 (20.4)	57 (22.3)	19 (14.0)
I	154 (27.4)		29 (22.3)	64 (29.1)	40 (30.5)	22 (25.9)	157 (27.4)		43 (23.8)	72 (28.1)	42 (30.9)
II	298 (53.0)		71 (54.6)	107 (48.6)	73 (55.7)	49 (57.6)	303 (52.9)		101 (55.8)	127 (49.6)	75 (55.1)
III	111 (19.6)		30 (23.1)	49 (22.3)	18 (13.7)	14 (16.5)	113 (19.7)		37 (20.4)	57 (22.3)	19 (14.0)
**Hormone receptor status**											
ER+	492 (87.1)	2	106 (81.5)	192 (86.9)	118 (90.8)	76 (90.5)	499 (87.2)	2	152 (84.4)	224 (87.2)	123 (91.1)
PgR+	394 (69.7)	2	81 (62.3)	151 (68.3)	98 (75.4)	64 (76.2)	399 (69.8)	2	119 (66.1)	179 (69.6)	101 (74.8)
ER+PgR+	390 (69.0)	2	80 (61.5)	149 (67.4)	97 (74.6)	64 (76.2)	395 (69.1)	2	118 (65.6)	177 (68.9)	100 (74.1)
ER+PgR-	102 (18.1)	2	26 (20.0)	43 (19.5)	21 (16.2)	12 (14.3)	104 (18.2)	2	34 (18.9)	47 (18.3)	23 (17.0)
ER-PgR-	69 (12.2)	2	23 (17.7)	27 (12.2)	11 (8.5)	8 (9.5)	69 (12.1)	2	27 (15.0)	31 (12.1)	11 (8.1)
ER-PgR+	4 (0.7)	2	1 (0.8)	2 (0.9)	1 (0.8)	0	4 (0.7)	2	1 (0.6)	2 (0.8)	1 (0.7)

*9 patients lacked genotype information.

**2 patients lacked genotype information.

***IQR Interquartile range.

### Early events in relation to IL6 diplotype

**Figure 1:**
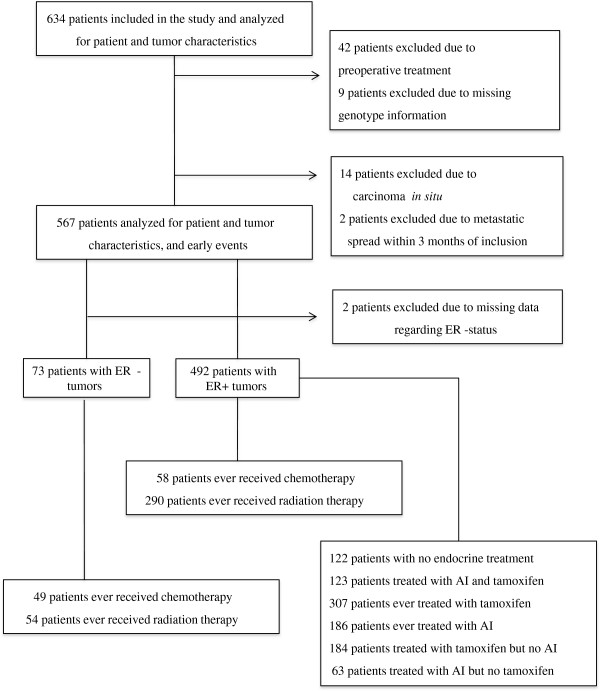
**Flow chart of the patient selection process.**

Patients who had received preoperative treatment (n = 42), who were diagnosed with carcinoma in situ (n = 14), and/or who had metastases detected earlier than three months after study inclusion (n = 2) were excluded from the survival analyses. A flowchart of the patients included and excluded in the analyses is presented in Figure 1. Among the 567 remaining patients, 86 were diagnosed with some type of breast cancer event (i.e., ipsi/contralateral, regional, or distant metastasis) during the 9-year follow-up time period; 54 of these patients had distant metastases. The median follow-up time was 5.1 years (IQR 3.0–7.1 years).

**Figure 2:**
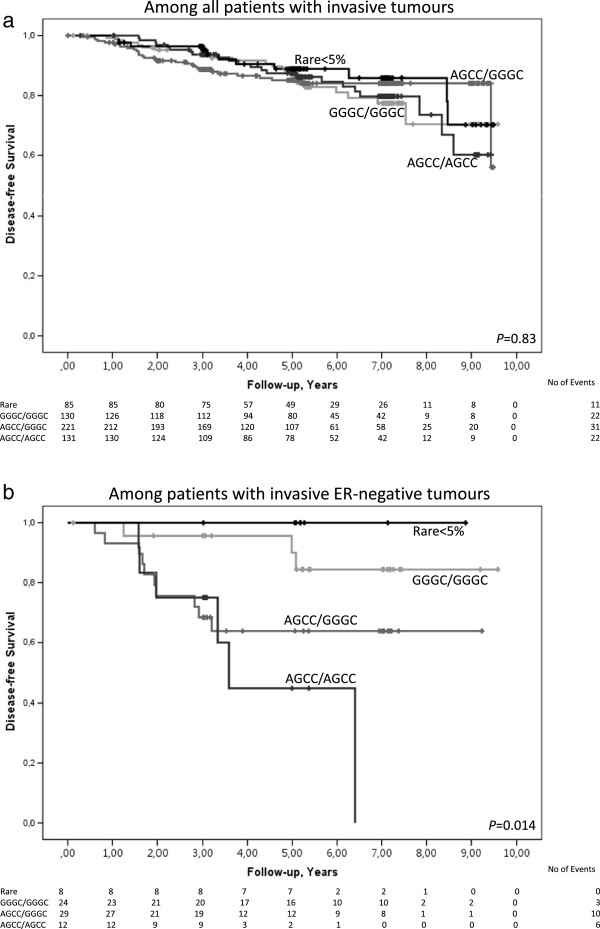
**Breast cancer-free survival in relation to** ***IL6*** **diplotype.** Kaplan-Meier estimates of breast cancer-free survival in relation to *IL6* diplotype. Since this is an ongoing cohort, there are fewer patients with longer follow-up times. **a)** Breast cancer-free survival among all patients with invasive tumours (Log Rank 3 df; *P* = 0.83). The adjusted HR was 1.14 (95% CI 0.62–2.10; *P* = 0.67). **b)** Breast cancer-free survival among patients with invasive ER-negative tumours (Log Rank 3 df; *P* = 0.014). The adjusted HR was 5.91 (95% CI 1.28–27.42; *P* = 0.023).

Among these 567 patients, *IL6* diplotype was not associated with early events in a univariable model (Figure 2a; Log Rank 3 df; *P* = 0.83) or in a multivariable model when adjusting for tumour size, axillary lymph node involvement, age, and histological grade III (adjusted HR = 1.14; 95% CI 0.62–2.10; *P* = 0.67). The addition of ER-status, BMI, WHR or breast volume to the model did not significantly change the results.

### Early events in relation to IL6 diplotype and ER-status

Among the 73 patients with ER-negative tumours, patients with the AGCC/AGCC or AGCC/GGGC genotypes had an increased risk of early events (Figure 2b; Log Rank 3 df; *P* = 0.014). In a multivariable model, this difference was only significant for the AGCC/AGCC diplotype (adjusted HR = 5.91; 95% CI 1.28–27.42; *P* = 0.023) compared to the GGGC/GGGC diplotype. When the dichotomous variable breast volume ≥850 ml was added to the model, the association between *IL6* AGCC/AGCC diplotype and risk of early events was strengthened (adjusted HR = 7.29; 95% CI 1.54–34.53; *P* = 0.012). The results remained essentially the same when BMI or WHR was added to the model.

Among the 492 patients with ER-positive tumours, the *IL6* diplotype was not associated with the risk of early events in a univariable model (Log Rank 3 df; *P* = 0.50) or in a multivariable model (adjusted HR = 0.73; 95% CI 0.37–1.45; *P* = 0.36). The addition of body constitution to the model did not essentially change the results.

### Early events in relation to IL6 diplotype, ER-status, and breast cancer treatment

Chemotherapy-treated patients with ER-negative tumours who had either an AGCC/GGGC or an AGCC/AGCC diplotype had an increased risk of early events (Log Rank 3 df; *P* = 0.031). This association was not observed among chemotherapy-treated patients with ER-positive tumours (Log Rank 3 df; *P* = 0.95). In a multivariable model of patients with ER-negative tumours who had received chemotherapy, patients with the AGCC/AGCC diplotype had an increased risk of early events compared to patients with the GGGC/GGGC diplotype (adjusted HR = 17.28; 95% CI 1.51–198.51; *P* = 0.022). The addition of body constitution to the model did not essentially change the results, except for the case of the dichotomous variable WHR > 85, which weakened the association (adjusted HR = 9.00; 95% CI 0.71–114.02; *P* = 0.090).

Radiotherapy-treated patients with ER-negative tumours who had either an AGCC/GGGC or an AGCC/AGCC diplotype had an increased risk of early events (Log Rank 3 df; *P* = 0.041). No such association was observed for radiotherapy-treated patients with ER-positive tumours (Log Rank 3 df; *P* = 0.58). In a multivariable model of patients with ER-negative tumours who had received radiotherapy, the association between the AGCC/AGCC diplotype and the risk of early events was borderline significant (adjusted HR = 5.71; 95% CI 0.98–33.33; *P* = 0.053). Similar results were obtained when body constitution was added to the model.

Among patients who had not received chemotherapy and patients who had not received radiotherapy, *IL6* diplotype was not associated with early events in any univariable or multivariable models.

### Early events in relation to the four individual IL6 SNPs

To investigate whether any of the SNPs were driving the results, each SNP was examined. No association was observed between rs1800796 and rs2069849 and risk of early events when stratifying according to ER-status or breast cancer treatment (data not shown). However, both rs1800795 Any C-carriers and rs1800797 Any A-carriers had an increased risk of early events among various subgroups of patients. Since rs1800795 and rs1800797 had an r^2^ = 0.966, we chose to continue our analyses with the most studied SNP: rs1800795. The following survival analyses were based on 574 patients as information on rs1800795 genotype was available for an additional seven patients with ER-positive tumours.

### Early events in relation to rs1800795

**Figure 3:**
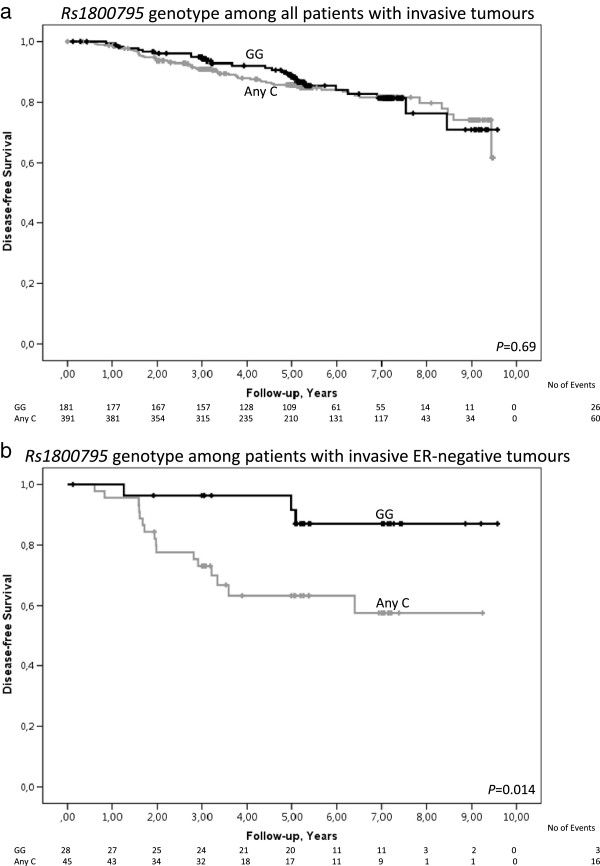
**Breast cancer-free survival in relation to rs1800795 genotype.** Kaplan-Meier estimates of breast cancer-free survival in relation to rs1800795 genotype. Since this is an ongoing cohort, there are fewer patients with longer follow-up times. **a)** Breast cancer-free survival among all patients with invasive tumours (Log Rank 1 df; *P* = 0.69). The adjusted HR was 1.11 (95% CI 0.69–1.77; *P* = 0.67). **b)** Breast cancer-free survival among patients with invasive ER-negative tumours (Log Rank 1 df; *P* = 0.014). The adjusted HR was 3.76 (95% CI 1.05–13.43; *P* = 0.041).

Among all patients, rs1800795 was not associated with early events in a univariable (Figure 3a; Log Rank 1 df; *P* = 0.69) or multivariable model (adjusted HR = 1.11; 95% CI 0.69–1.77; *P* = 0.67). Adjusting for ER-status or body constitution generated similar results.

### Early events in relation to rs1800795 and ER-status

Among patients with ER-negative tumours, Any C-carriers had an increased risk of early events compared to GG-carriers (Figure 3b; Log Rank 1 df; *P* = 0.014). This difference was also observed using a multivariable model (adjusted HR = 3.76; 95% CI 1.05–13.43; *P* = 0.041). The results were essentially the same when body constitution was added to the model.

Among patients with ER-positive tumours, rs1800795 was not associated with early events in a univariable model (Log Rank 1 df; *P* = 0.38) or in a multivariable model (adjusted HR = 0.79; 95% CI 0.47–1.33; *P* = 0.37). The addition of body constitution to the model yielded similar results.

### Early events in relation to rs1800795, ER-status, and breast cancer treatment

**Figure 4:**
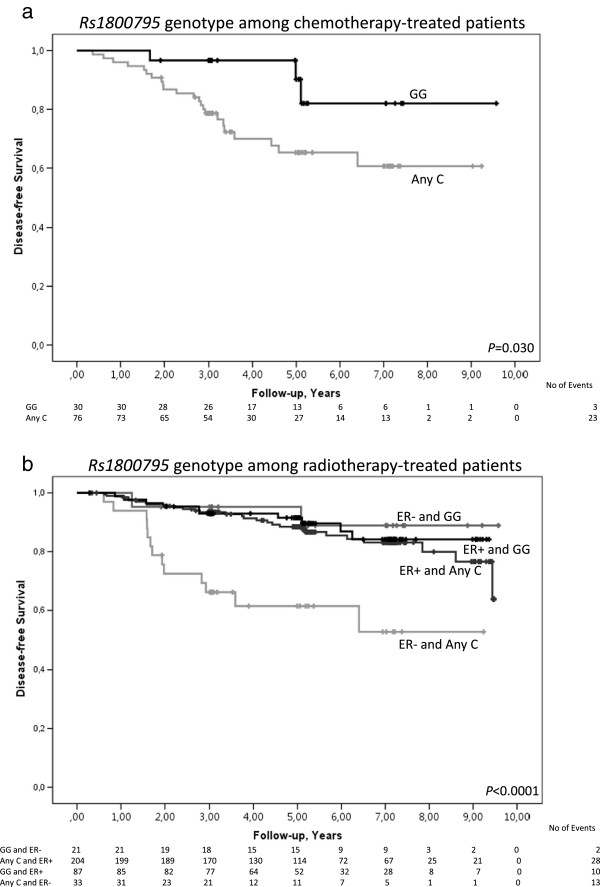
**Breast cancer-free survival in different treatment groups in relation to rs1800795 genotype. a)** Kaplan-Meier estimates of breast cancer-free survival in relation to rs1800795 genotype among chemotherapy-treated patients (Log Rank 1 df; *P* = 0.030). The adjusted HR was 3.42 (95% CI 1.01–11.54; *P* = 0.048). Since this is an ongoing cohort, there are fewer patients with longer follow-up times. **b)** Kaplan-Meier estimates of breast cancer-free survival in relation to rs1800795 genotype among radiotherapy-treated patients (Log Rank 3 df; *P* < 0.0001). The adjusted HR was 7.17 (95% CI 1.16–32.28; *P* = 0.010). Since this is an ongoing cohort, there are fewer patients with longer follow-up times.

Irrespective of ER-status, chemotherapy-treated Any C-carriers had an increased risk of early events compared to GG-carriers (Figure 4a; Log Rank 1 df; *P* = 0.030). This difference was also observed using a multivariable model (adjusted HR = 3.42; 95% CI 1.01–11.54; *P* = 0.048). When the dichotomous variable breast volume ≥850 ml was added to the model, the association between being an Any C-carrier and having an increased risk of early events was strengthened (adjusted HR = 3.71; 95% CI 1.08–12.75; *P* = 0.037). The addition of ER-status or other body constitution-related parameters to the model did not essentially change the results.

Radiotherapy-treated Any C-carriers with ER-negative tumours had an increased risk of early events compared to GG-carriers (Figure 4b; Log Rank 3 df; *P* < 0.0001). This finding was also observed using a multivariable model (adjusted HR = 7.17; 95% CI 1.16–32.28; *P* = 0.010). Similar results were obtained when body constitution was added to the model. No such association was observed among radiotherapy-treated Any C-carriers with ER-positive tumours.

Among patients who had not received chemotherapy and patients who had not received radiotherapy, rs1800795 was not associated with early events in any univariable or multivariable models.

### Early events in relation to rs1800795 and antidepressant treatment

At the preoperative visit, 56 of the patients included in the survival analyses were undergoing some type of prescribed antidepressant treatment. Three of these patients ceased treatment during the first year after their breast cancer diagnosis. During the first year after diagnosis, 23 additional patients started antidepressant treatment. Any C-carriers (n = 383) were more likely to have started than to have ceased antidepressant treatment during the first year after their breast cancer diagnosis (McNemar’s test *P* = 0.001). For GG-carriers (n = 176), this association was not significant (McNemar’s test *P* = 0.11); however, no significant differences were observed between Any C-carriers and GG-carriers.

No association was observed between antidepressant treatment prior to any breast cancer event and risk of early events, either at the preoperative visit (Log Rank 1 df; *P* = 0.61) or during any visit up to the 1-year visit (Log Rank 1 df; *P* = 0.32). Multivariable models yielded similar results.

## Discussion

The present study investigated the impact of *IL6* genotype on early events and treatment response in an ongoing cohort of breast cancer patients. The main finding was that *IL6* genotype was strongly associated with early events among patients with ER-negative tumours, especially in radiotherapy-treated patients, and among chemotherapy-treated patients irrespective of ER-status. To our knowledge, the association of *IL6* genotype with disease-free survival and treatment response in relation to tumour ER-status has not been previously investigated in a large population-based cohort of breast cancer patients.

The high-risk AGCC/AGCC diplotype contains the high-risk genotypes of the SNPs rs1800795 and rs1800797. The association between risk of early events and diplotype appears to be primarily driven by the SNPs rs1800795 and rs1800797, as no association was observed between the SNPs rs1800796 and rs2069849 and disease-free survival. Therefore, the discussion will focus primarily on the association between rs1800795 and disease-free survival.

Some studies reported that the rs1800795 C-allele was associated with reduced systemic levels of IL-6 [16, 17]. However, several studies found that the lower levels of IL-6 that were observed among C-allele carriers compared to G-allele carriers were increased and surpassed the levels observed in G-allele carriers when both genotypes were subjected to inflammatory stimuli [14, 33, 34]. Therefore, we hypothesise that the C-allele is associated with increased levels of IL-6; however, this association only exists in the presence of inflammatory stimuli, such as ER-negativity, radiotherapy or chemotherapy. A tentative explanation for the association between *IL6* genotype and inflammatory stimuli might be found in a differential reduction in the methylation of the *IL6* promoter, because rs1800795 has been reported to be in linkage with the rs2069845 CpG site [35].

In the present study, *IL6* genotype predicted disease-free survival only among patients with ER-negative tumours, unless the patients had received chemotherapy. A previous study demonstrated that IL-6 constitutively activates the NF-κβ pathway, which subsequently drives further IL-6 production, creating a positive feedback loop [36]. As reviewed by Shostack *et al*., NF-κβ plays a crucial role in breast cancer progression by stimulating proliferation and preventing apoptosis. NF-κβ is more frequently constitutively activated in ER-negative tumours than in ER-positive tumours, and ERα has been shown to repress NF-κβ activation [37]. Further, NF-κβ inhibition has been shown to inhibit proliferation of ER-negative cells [38]. If Any C-carriers with ER-negative tumours have an increased level of IL-6, an associated increase in NF-κβ may explain the impaired prognosis among those patients.

Among patients who had received chemotherapy, Any C-carriers had an over 3-fold increased risk of events compared to GG-carriers, regardless of their ER-status. Chemotherapy has been shown to increase IL-6 levels in breast cancer patients and to activate NF-κβ [39, 40]. IL-6 levels and NF-κβ activation are associated with chemotherapy resistance, and inhibition of NF-κβ has sensitised chemotherapy resistant cell lines to chemotherapy [6, 41]. The increased levels of IL-6 in Any C-carriers that occur in response to chemotherapy-associated inflammation and the related increase in NF-κβ could explain the elevated risk of events among chemotherapy-treated Any C-carriers that was observed in the present study. Inhibitors of NF-κβ or IL-6 could be of interest for this subgroup of patients.

In the present study, radiotherapy-treated Any C-carriers with ER-negative tumours had an over 7-fold increased risk of events compared to GG-carriers. IL-6 levels and NF-κβ activation have been linked to radiotherapy resistance [6]. In addition, in a randomised control trial, radiotherapy conferred significantly smaller improvements in recurrence control for patients with ER-negative tumours than for patients with ER-positive tumours [42]. A majority of the early events that occurred among Any C-carriers with ER-negative tumours were distant metastases (9 out of 13 events). Hence, the increased risk of this group cannot be explained by local radiotherapy resistance. Radiation induces inflammatory signalling, which lasts long beyond the initial radiation-induced burst [43]. This signalling is mainly executed via the activation of NF-κβ, but IL-6 levels have also been shown to increase [44]. According to our previously stated hypothesis, radiotherapy-induced inflammation would be particularly harmful for Any C-carriers with ER-negative tumours, as this inflammation represents an additional inflammatory stimulus that induces Any C-carriers to release large amounts of systemic IL-6, explaining the impaired prognosis among those patients. Inhibitors of NF-κβ or IL-6 could be of interest for this subgroup of patients; previous studies reported promising results after treating radiotherapy resistance with NF-κβ inhibitors [44].

The addition of body constitution to the multivariable models modified the association between *IL6* genotype and disease-free survival in some subgroups. IL-6 levels and body constitution are closely linked. Body constitution-related parameters, such as obesity, a high WHR, and a large breast volume, are associated with breast cancer prognosis [27, 45, 46]. This association is believed to be mediated by chronic inflammation [47]. Our group recently reported that the inflammation-associated *COX2* rs5277 SNP impacted breast cancer prognosis differently depending on tumour ER-status, type of breast cancer treatment, and body constitution [48]. However, no association was observed between rs5277 on chromosome 1 and rs1800795 on chromosome 7 (data not shown). Hence, the findings of the present study are independent from the previous study.

Although no association was observed between antidepressant use and genotype in the present study, the relationship between genotype and depression could not be assessed using our questionnaire. Depression is estimated to be underdiagnosed in cancer patients [49]. To properly investigate the association between genotype and depression, it would be necessary to collect additional information on depressive symptoms.

The results of the present study are in contrast to the results of two previous studies [8, 10], in which chemotherapy-treated GG-carriers with ER-positive tumours had a shorter disease-free survival than Any C-carriers. However, these studies investigated this association in two smaller cohorts of chemotherapy-treated patients. One cohort had a median axillary lymph node involvement of 14 nodes, and in the other cohort, 81% of patients had ≥10 positive lymph nodes. The present study and these previous studies are not comparable, because our cohort comprises a population-based series of breast cancer patients in which the majority of patients were node negative.

The present study did not have access to systemic IL-6 levels. However, intrapersonal IL-6 levels vary greatly depending on the time of day, food intake, recent exercise routines, et cetera [50]. Stable genomic information on inflammatory tendency may be superior for assessing ongoing systemic inflammation, as it is not affected by daily fluctuations. This study is population-based, and the included patients were similar to non-included patients with respect to age and hormone receptor status [22]. SNP genotyping is considered reliable in the present study, as over 10% of the samples were run in duplicate, with a concordance of 100%. In the present study, the vast majority of patients were ethnic Swedes. Studies of the association between *IL6* genotype and breast cancer prognosis in other ethnic groups are warranted.

Since the median follow-up time was only 5 years, the long-term effects of *IL6* genotype on disease-free survival could not be evaluated. The estimated failure rates in the previous power calculation exceeded the observed failure rates; these failure rates may have influenced the power of this study. In addition, this study assessed a number of variables and some of the findings may be attributable to chance. The results must be confirmed in independent cohorts.

## Conclusions

The main finding of the present study was that *IL6* genotype was strongly associated with early events among patients with ER-negative tumours, particularly among radiotherapy-treated patients, and among chemotherapy-treated patients irrespective of ER-status. We hypothesise that the mechanism that underlies this observation is that the rs1800795 C-allele is associated with increased systemic levels of IL-6 in the presence of inflammatory stimuli, such as ER-negativity, radiotherapy, and chemotherapy. The high risk for early events observed in certain subgroups of patients suggests that combined information on *IL6* genotype, tumour ER-status, and breast cancer treatment may represent a tool for identifying patients who require more personalised treatment, potentially using inhibitors of NF-κβ or IL-6.

## Electronic supplementary material

Additional file 1: **Patient characteristics for all patients, and for the patients included in the survival analyses.** (XLSX 244 KB)

Additional file 2: **Tumour characteristics for all patients, and for the patients included in the survival analyses.** (XLSX 16 KB)

## Abbreviations

BMI: *Body mass index*
CRP: *C-reactive protein*
DNA: *Deoxyribonucleic acid*
ER: *Oestrogen receptor*
IL-6: *Interleukin-6*
*IL6*: *Interleukin-6 gene*
IQR: *Interquartile range*
ln: *Natural logarithm*
PgR: *Progesterone receptor*
SNP: *Single nucleotide polymorphisms*
WHR: *Waist-to-hip ratio*.
